# Effects of the sucrose concentrations and incubation periods on in vitro pollen germination and pollen tube growth in three rice cultivars

**DOI:** 10.5511/plantbiotechnology.24.1017a

**Published:** 2025-03-25

**Authors:** Neema Yona Yohana, Arisa Nakano, Yoichiro Hoshino

**Affiliations:** 1 Division of Biosphere Science, Graduate School of Environmental Science, Hokkaido University, Kita 11, Nishi 10, Kita-ku, Sapporo, Hokkaido 060-0811, Japan; 2 Field Science Center for Northern Biosphere, Hokkaido University, Kita 11, Nishi 10, Kita-ku, Sapporo, Hokkaido 060-0811, Japan

**Keywords:** in vitro pollen germination, incubation period, *Oryza sativa*

## Abstract

High-quality pollen grains are essential for artificial cross pollination and grain production. The optimization of culture conditions for in vitro pollen germination is useful for evaluating pollen quality. However, there is limited information on in vitro pollen germination system for rice (*Oryza sativa* L.). Therefore, this study aimed to develop an efficient pollen germination system for rice and determine the optimal incubation period, incubation temperature, and sucrose concentration. Three rice cultivars were studied: ‘Nanatsuboshi’, ‘Nipponbare’, and ‘Kitaake’ and culture media developed in the previous study were used to optimize the conditions. The highest pollen germination rates for all cultivars were observed in the medium containing 20% (w/v) sucrose. Pollen tube bursting was observed during pollen tube elongation. We discussed the relationship between the incubation period and pollen tube bursting. This study contributes to evaluating rice pollen germination, pollen tube growth, and pollen tube bursting to support grain production.

## Introduction

Pollen quality assessment is necessary for successful fertilization and can improve crop yield and quality. Pollen quality can be assessed in vitro by measuring pollen viability, which includes pollen germination, pollen tube elongation, and pollen tube bursting (Franklin-Tong 2010; Lange and Wojciechowska 1976). The importance of pollen quality analysis for better genetics and productivity gains of rice has created a strong demand for optimizing the germination medium for rice pollen, by which high pollen germination and pollen tube growth can be assessed accurately in vitro. Pollen germination and pollen tube growth in vitro in artificial germination media, depends on media components and composition. Rice pollen is highly sensitive to the pollen germination medium components and composition (Dafni and Firmage 2000). Similar to most crop pollen grains, rice pollen requires sugar to germinate and develop into the pollen tubes (Zhou et al. 2020). Sucrose is the preferred source of carbohydrates for supporting pollen germination and pollen tube growth in vitro (Lagera et al. 2017; Montaner et al. 2003; Sarkar et al. 2018; Tushabe and Rosbakh 2021). The major role of sucrose is to regulate the osmotic pressure between the medium and pollen grains and to provide energy during pollen germination and pollen tube growth (Chen et al. 2023; Hirsche et al. 2017). Temperature has effects on pollen development during reproductive stage (Matsui et al. 2001). Several studies reported that incubation temperature can affect the pollen germination and pollen tube growth rates in vitro (Acar and Kakani 2010; Wang et al. 2000).

Research on the optimization of media and germination conditions in vitro for the high reproducibility of pollen germination and pollen tube growth has been reported in several crops such as petunia (Hoshino et al. 2016), sorghum (Lansac et al. 1994), wheat and other 86 plant species (Brewbaker and Kwack 1963; Tian et al. 1998), tomato and pepper (Langedijk et al. 2023), Ester lily and tobacco (Sawidis 2008) and rice (Khatun and Flowers 1995; Wang et al. 2000). Khatun and Flowers (1995) used stains and in vitro germination method to test pollen viability. Wang et al. (2000) investigated the medium composition, focusing on pollen germination and pollen tube growth. Based on the medium developed by Wang et al. (2000) with minor modification, we examined pollen germination of Japanese rice cultivars. Among these Japanese cultivars, ‘Nipponbare’ and ‘Kitaake’ have been used for genomic studies as the model varieties (Jain et al. 2019; Li et al. 2017). ‘Nanatsuboshi’ developed by the anther culture method at Hokkaido Central Agricultural Experiment Station retains elite characteristics such as high-yielding, strong cold tolerance, and blast resistance (Yoshimura et al. 2002). In this study, we used these specific 3 rice cultivars to analyze pollen germination behaviors. The data obtained in this study will be utilized for further research on genetic and molecular levels in ‘Nipponbare’ and ‘Kitaake’, and for evaluating pollen quality in ‘Nanatsuboshi’ at cold regions.

This study aimed to determine the optimal sucrose concentration, incubation period, and incubation temperature for pollen of three Japanese rice cultivars by conducting a pollen viability assessment. We established procedures to optimize rice pollen germination conditions for better pollen viability assessments in vitro. The results of this study will help determine the pollen germination capacity, pollen tube growth rate, and pollen tube bursting rates in rice in vitro.

## Materials and methods

### Plant materials

The seeds of rice (*Oryza sativa* L.) cultivars ‘Nanatsuboshi’, ‘Kitaake’, and ‘Nipponbare’, were collected from the Plant Breeding Laboratory, Hokkaido University, Japan. The seeds were surface-sterilized with a 5% sodium hypochlorite (NaClO) solution with two drops of polyoxyethylene sorbitan monolaurate (Tween 20), rinsed thoroughly with distilled water, and germinated on a Petri dish in the laboratory. The seeds were surface-sterilized to treat any seedborne disease before seedling establishment. Seven days after germination, seedlings were transferred to seedling-growing trays for 14 days. Then, they were transferred to pots with a manure-soil mix in the greenhouse, and fertilizer was applied to maintain seedling health. Watering was done in the morning and evening. The greenhouse was managed with natural temperature; when the greenhouse temperature exceeded 25°C, the roof vent was opened during the summer season. The greenhouse temperate was maintained at 25°C by the heating system during the winter season.

### Pollen germination in vitro

A liquid culture medium was prepared and used for pollen germination, according to a report by (Hirano and Hoshino 2009; Wang et al. 2000), with minor modifications. The basal medium contained 0.01% (w/v) CaNO_3,_ 0.01% (w/v) H_3_BO_3,_ 0.0007% (w/v) KH_2_PO_4_, 10% (w/v) PEG4000, and 0.01% (w/v) yeast extract. Four media containing different sucrose concentrations (0, 10, 20 and 25% (w/v)) were prepared. In the medium containing 0% sucrose (control), the PEG4000 was 0%. The media were adjusted to pH 5.8 by either adding 0.01 N potassium hydroxide (KOH) solution or 0.01 N hydrochloric acid (HCl) solution. After pH adjustment, the media were sterilized by autoclaving at 121°C for 15 min and maintained at 5°C until use.

**Figure 1:**
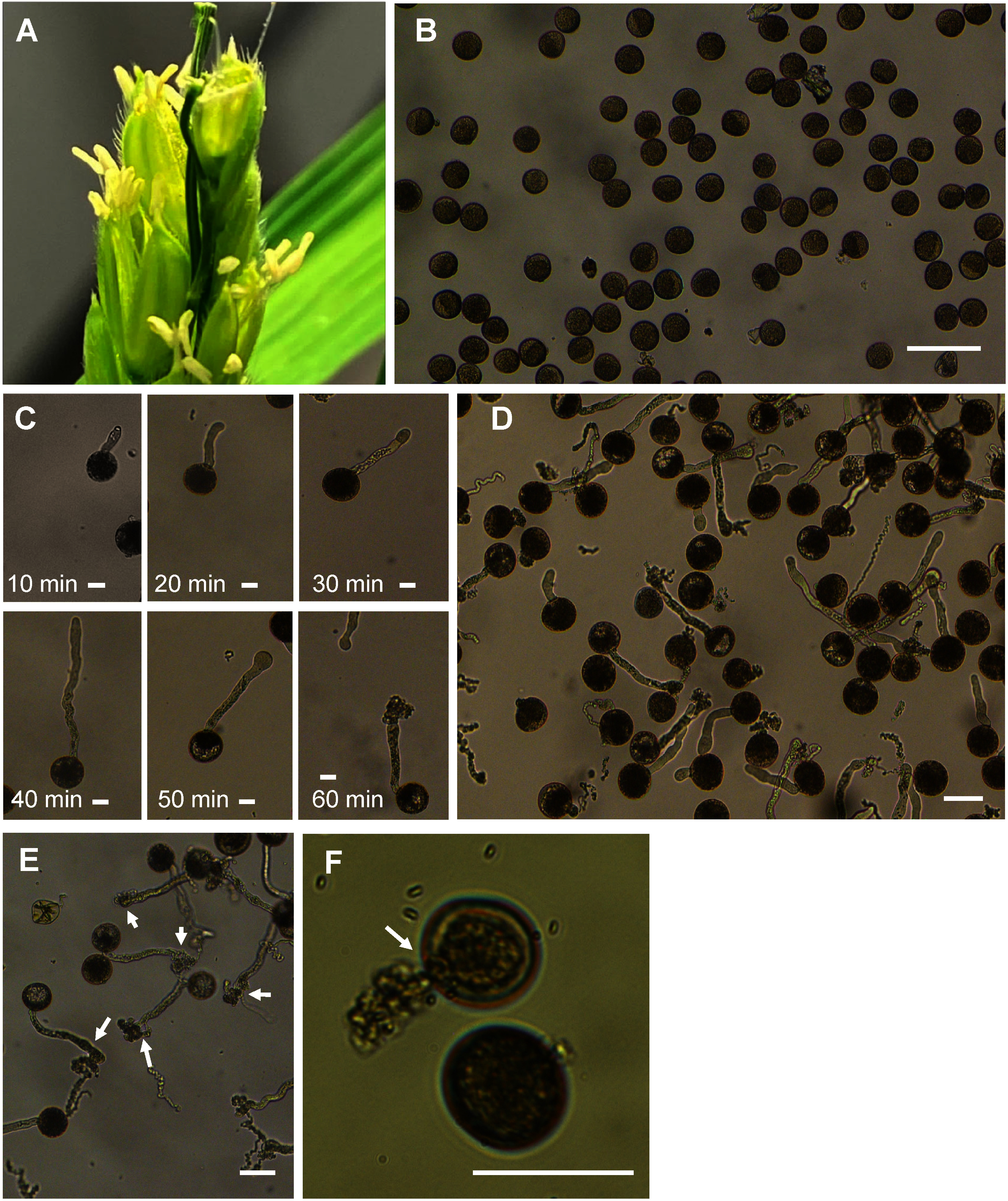
Figure 1. Rice pollen germination progressed from anthesis to pollen tube bursting. (A) Anthesis stage at which pollen was sampled. (B) Pollen grains are plated on a Petri dish at very early five min (no germination initiation). (C) Progressive results of pollen germination, pollen tube growth, and pollen tube bursting from 10 to 60 min. (D) A representative image of pollen germination under an inverted microscope. (E) Bursting of pollen tubes (arrows). (F) Bursting of pollen grain (arrow). Bar for B=100 µm, C=20 µm, D, E, and F=50 µm.

Fresh mature pollen grains were sampled at 10:30 A.M. from rice flowers (Figure 1A) during the anthesis stage by shaking the flowers directly on a Petri dish containing a liquid medium and examining for pollen grains (Figure 1B). The pollen grains on all four media were cultured in a 35 mm×10 mm Petri dish with 2.0 ml liquid culture medium at 25°C in dark conditions. The germinated pollen grains were observed at 10, 20, 30, 40, 50, and 60 min of incubation to record the biological changes in pollen grains (Figure 1C). For determining the pollen germination rate, at least a total of 100 pollen grains were counted per microscopic fields of views in each media using an inverted microscope (Axiovert 200; Zeiss, Oberkochen, Germany) and observed both the germinated and ungerminated pollen grains (Figure 1D). Pollen grains were considered germinated if the pollen tube length was equal to or greater than the pollen grain diameter and the pollen germination rate (%) was determined by dividing the number of germinated pollen grains by the total number of observed pollen grains.

### Plasmolysis observation

Pollen grains were evaluated for plasmolysis as effects of sucrose concentrations in culture medium during pollen germination and pollen tube growth. Pollen samples for ‘Nanatsuboshi’ were incubated in culture media containing different sucrose concentrations (0, 10, 20 and 25%). Plasmolysis was observed during the culture period.

### Pollen tube bursting observation

Pollen tube bursting was observed within 60 min of incubation. Burst pollen tubes (Figure 1E) and burst pollen grains (Figure 1F) were observed. The number of burst pollen tubes was counted and recorded per microscopic field of views in the media using an inverted microscope. The pollen tube bursting rates (%) were calculated by dividing the number of pollen tube burst by the total number of pollen grains germinated per microscopic field of view. Percentage values were used for the statistical analysis.

### FDA staining test

The viability of pollen grains was assessed with fluorescein diacetate (FDA) according to the protocol of Hoshino et al. (2006) with minor modifications. A working solution was prepared at 10 µg ml^−1^ with the culture medium containing 20% (w/v) sucrose. The samples were observed using an epifluorescence microscope (Axiovert 200). The fluorescence was detected with the excitation filter set to 17. Images were taken with a camera (DS-Fi2-L3; Nikon, Tokyo, Japan). The frequency of FDA-stained pollen grains was compared with the pollen germination rates in each cultivar pollen grains taken from the same plants at the same day.

### Effects of incubation temperatures on pollen germination

Incubation temperatures were examined at 25, 27 and 30°C for pollen germination in 20% (w/v) sucrose-containing medium. The pollen grains of ‘Nanatsuboshi’ were used. Pollen germination was evaluated. The experiments were repeated three times.

### Statistical analysis

A one-way analysis of variance was used to test the statistical differences between the means of the independent groups (rice cultivars) and the mean differences among the treatments (same cultivar but different incubation periods and temperature). Means were separated using Duncan’s multiple range test at the 5% level (*p*≤0.05). Percentage data were normalized by angular (arcsine) transformation before statistical analysis (Steel and Torrie 1960). Three replicates were used to assess sucrose concentrations and incubation temperatures, and five replicates were used to evaluate the incubation periods. The data analysis and the means between FDA and in vitro pollen germination were done and compared using Student’s *t*-test at *p*≤0.05.

## Results

### Effects of sucrose concentration on pollen germination

**Figure 2:**
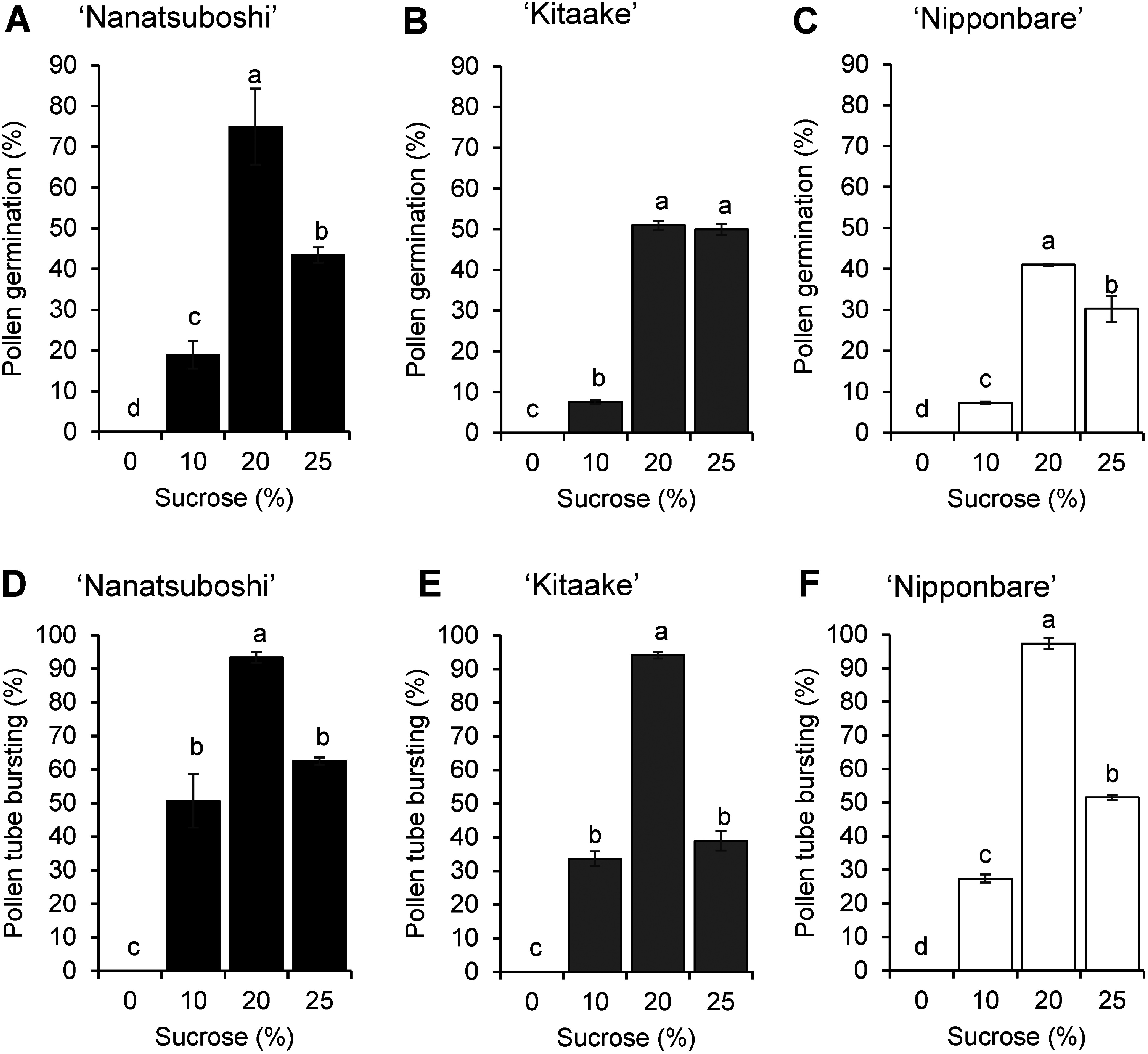
Figure 2. Effect of sucrose concentrations on pollen germination, and pollen tube bursting in rice cultivars. Pollen germination rate in (A) ‘Nanatsuboshi’, (B) ‘Kitaake’, and (C) ‘Nipponbare’. Pollen tube bursting rate in (D) ‘Nanatsuboshi’, (E) ‘Kitaake’, and (F) ‘Nipponbare’. The results are the average of incubation periods of 60 min for three replicates. Values followed by the same letters are not significantly different at *p*≤0.05, according to Duncan’s multiple range test. Data represent means±SE (*n*=3).

The effects of different sucrose concentrations (0, 10, 20 and 25%) on in vitro pollen germination rate, and pollen bursting rate were evaluated 60 min after culture. In all cultivars, significant differences (*p*≤0.05) were observed in pollen germination rate, and pollen tube bursting rate in different sucrose concentrations (Figure 2). The highest pollen germination rates, and pollen tube bursting rates were observed in 20% sucrose medium in all rice cultivars (Figure 2). Among the three cultivars examined, ‘Nanatsuboshi’ exhibited the highest pollen germination rate, reaching 74.96% (Figure 2A), followed by ‘Kitaake’ at 50.99% (Figure 2B), and ‘Nipponbare’ at 41.03% (Figure 2C). These rates of 74.96, 50.99 and 41.03% germination were observed in response to varying sucrose concentrations. The pollen tube bursting rates were higher than 90% in the 20% sucrose medium in all cultivars (Figure 2D–F).

### Effects of incubation period on pollen germination

**Figure 3:**
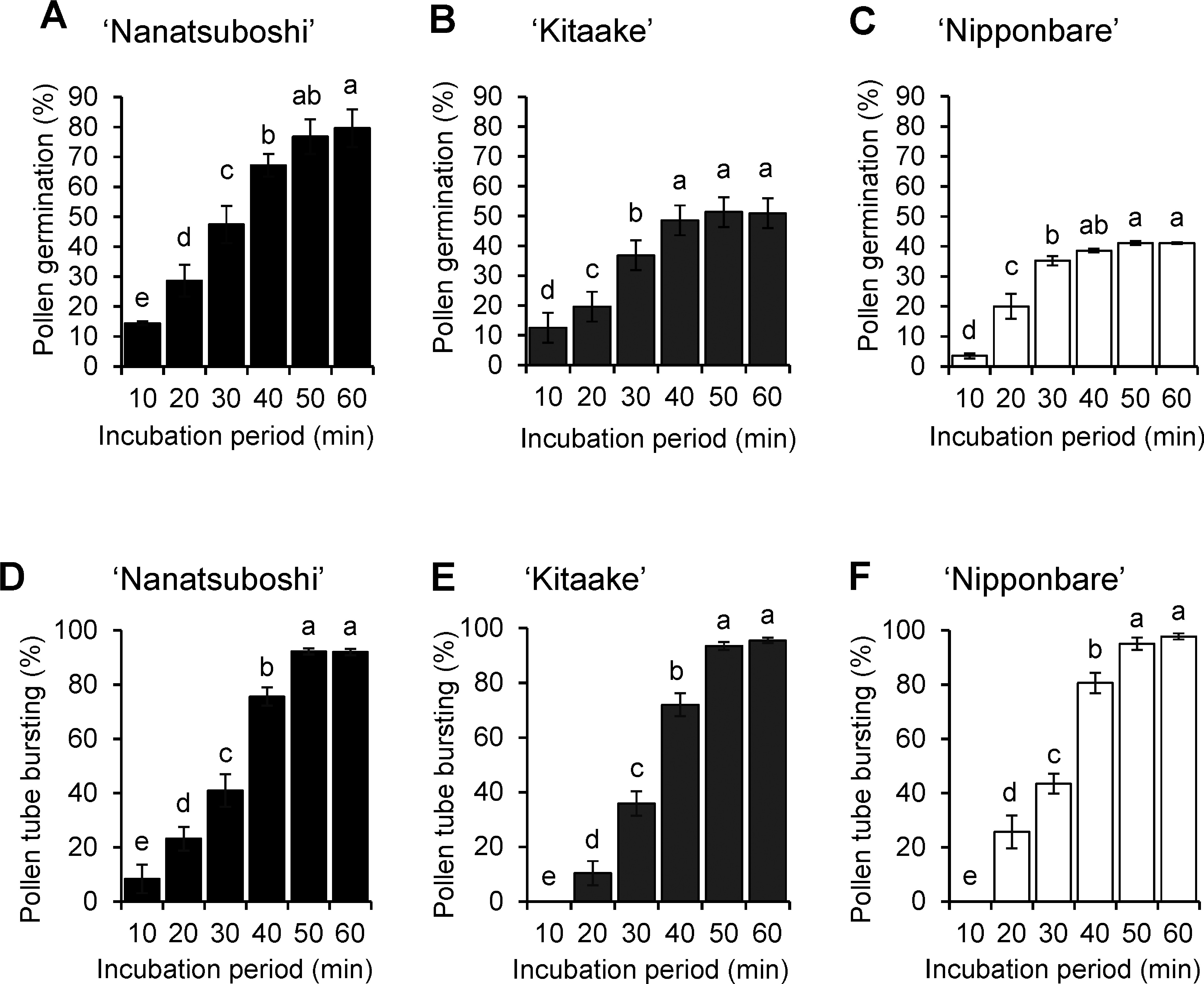
Figure 3. Effects of incubation periods on pollen germination, and pollen tube bursting in rice cultivars. Pollen germination rate in (A) ‘Nanatsuboshi’, (B) ‘Kitaake’, and (C) ‘Nipponbare’. Pollen tube bursting rate in (D) ‘Nanatsuboshi’, (E) ‘Kitaake’, and (F) ‘Nipponbare’. The medium including 20% sucrose was used. Values followed by the same letters are not significantly different at *p*≤0.05, according to Duncan’s multiple range test. Data represent means±SE (*n*=5).

The effects of incubation period on in vitro pollen germination were evaluated in the three rice cultivars. The results showed that the pollen germination rates increased along with the incubation time in all cultivars (Figure 3). In ‘Nanatsuboshi’, the pollen germination rates were maximized at 50 to 60 min incubation periods (Figure 3A). The pollen germination rates became stationary at 40 to 60 min incubation periods (Figure 3B, C) in ‘Kitaake’ and ‘Nipponbare’. In these experiments, ‘Nanatsuboshi’ showed the highest pollen germination rate among the three cultivars.

The statistical analysis indicated that there were significant differences (*p*≤0.05) in pollen tube bursting rate for different incubation periods from 10 to 50 min for all rice cultivars (Figure 3D–F). The results revealed that the pollen tube bursting rates were increased over the incubation period for all three cultivars. In all cultivars, about 90% of pollen tubes showed bursting at 60 min of the incubation period.

### FDA staining test

**Figure 4:**
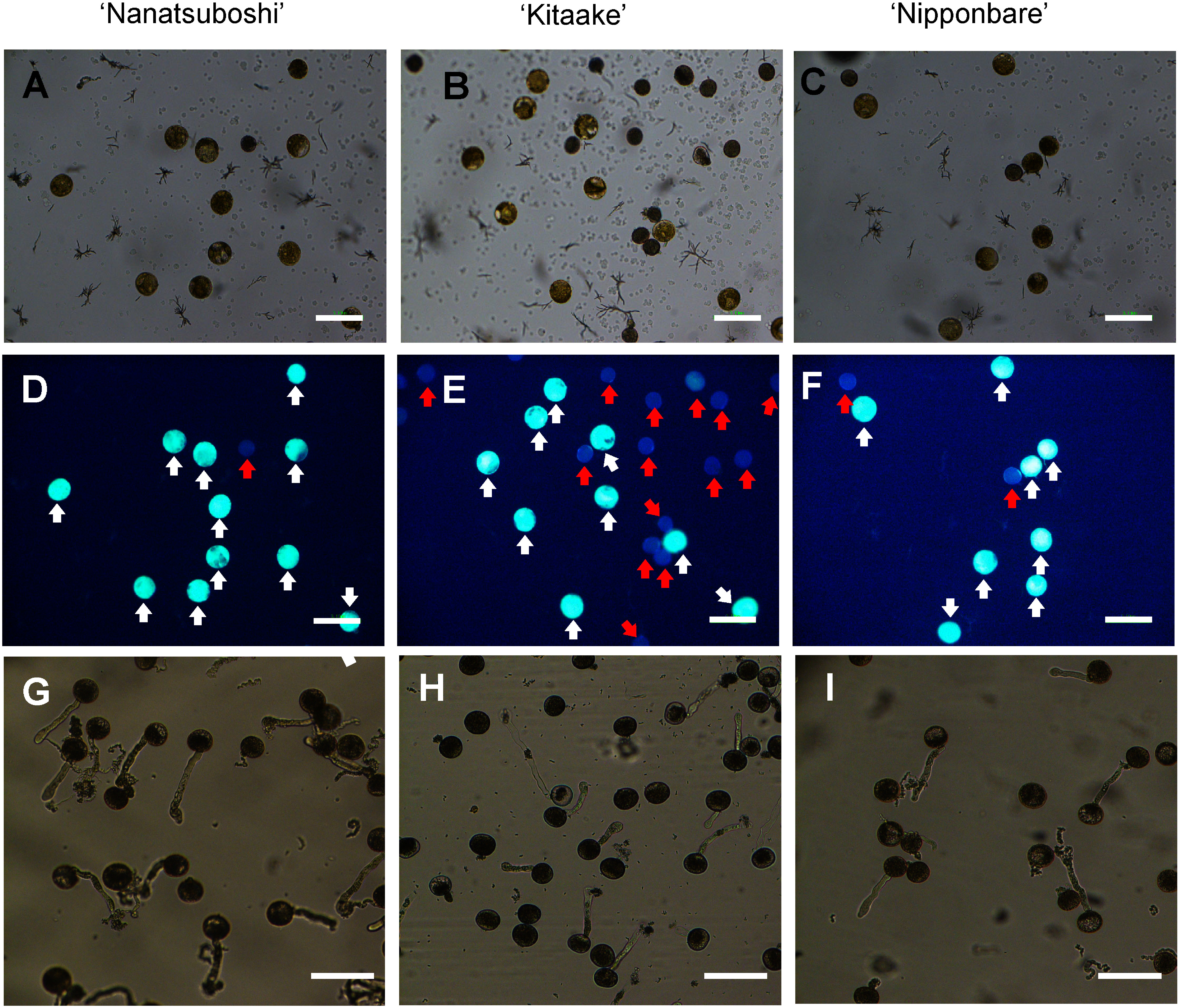
Figure 4. Comparison of FDA staining and pollen germination in vitro for three rice cultivars. Microscopic images of (A) ‘Nanatsuboshi’, (B) ‘Kitaake’, and (C) ‘Nipponbare’ pollen grains. FDA staining of (D) ‘Nanatsuboshi’, (E) ‘Kitaake’, and (F) ‘Nipponbare’ pollen grains. The images of (D–F) were taken from the same samples as (A–C). White arrows and red arrows indicate FDA-stained pollen grains and FDA negative pollen grains, respectively. Pollen germination of (G) ‘Nanatsuboshi’, (H) ‘Kitaake’, and (I) ‘Nipponbare’. Bars=100 µm.

The viability test with FDA staining (Figure 4A–F and Table 1) was conducted to compare with in vitro germination rates (Figure 4G–I and Table 1) in the three rice cultivars. FDA-stainability were higher than pollen germination rate in all cultivars. The highest FDA stainability and pollen germination rate were obtained in ‘Nanatsuboshi’ followed by ‘Kitaake’ and ‘Nipponbare’. The tendency between FDA stainability and pollen germination rate was consistent in all cultivars.

**Table 1:** Table 1. The percentages of FDA-stained pollen grains and pollen germination in three rice cultivars.

Rice cultivar	FDA-stainability (%)	Pollen germination rate (%)	Significance
‘Nanatsuboshi’	92.85±0.96	80.36±0.69	*
‘Kitaake’	76.41±1.28	60.73±1.38	*
‘Nipponbare’	74.11±1.64	54.87±0.64	*

* Statistical significance level: *p*≤0.05. Significances of means between the FDA-staining test and pollen germination were analyzed using Student’s *t*-test. The values are the percentage means±SE. SE stands for standard error of means.

### Plasmolysis was observed at different sucrose concentrations

**Figure 5:**
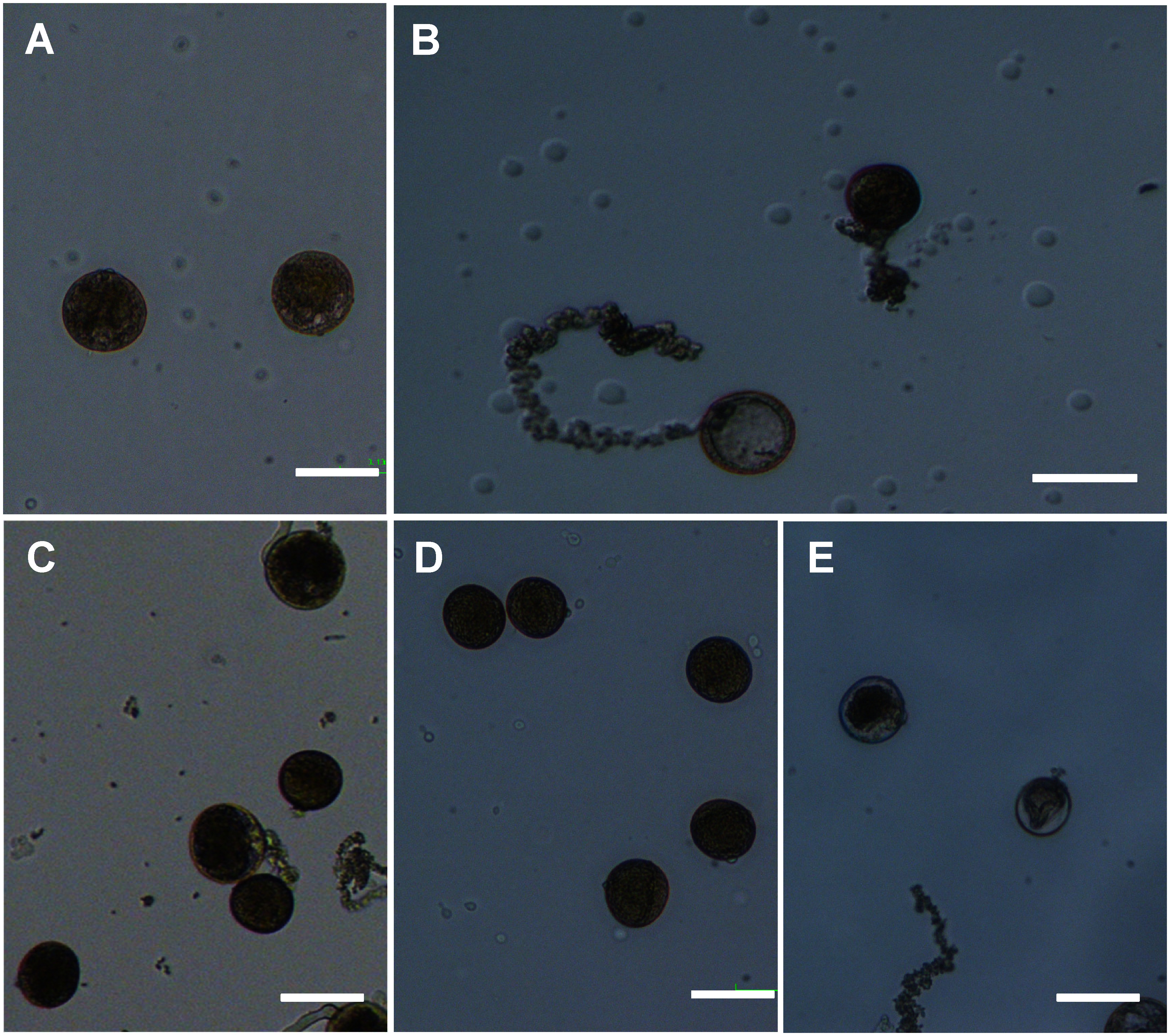
Figure 5. Plasmolysis observation of pollen grains in different sucrose concentrations in ‘Nanatsuboshi’ rice cultivar. (A) Swelling of pollen grains in 0% sucrose. (B) Bursting of pollen grains in 0% sucrose. (C) Enlargement of pollen grains in 10% sucrose. (D) Consistent shape of pollen grains in 20% sucrose. (E) Plasmolysis of pollen grains in 25% sucrose. Bars=50 µm.

Plasmolysis of pollen grains was observed in the culture media with different sucrose concentrations (0, 10, 20, and 25%) during the culture period. In 0% sucrose, the pollen grains became swell (Figure 5A) and then showed bursting (Figure 5B). In 10% sucrose, the pollen grains became to be large (Figure 5C). In 20% sucrose, the shape of pollen grains was kept (Figure 5D). In 25% sucrose, plasmolysis of pollen grains was obvious (Figure 5E). Pollen germination was observed in 10, 20 and 25% sucrose-containing medium as shown in Figure 2.

### Effects of incubation temperatures on pollen germination in vitro

The effect of incubation temperatures was evaluated at 25, 27 and 30°C for 60 min in ‘Nanatsuboshi’ (Table 2). Pollen germination was observed at 25 and 27°C. No pollen germination was shown at 30°C. There were no significant differences in pollen germination rate between 25 and 27°C.

**Table 2:** Table 2. Effects of incubation temperatures on pollen germination in ‘Nanatsuboshi’ rice cultivar.

Incubation temperature	Pollen germination rate (%)
25°C	85±0.87^a^
27°C	80±1.17^a^
30°C	0±0.00^b^

The values are means for pollen germination rates (%)±SE. The values with the same letter(s) in a column are not significantly different at *p*≤0.05 according to Duncan’s multiple range test.

## Discussion

In this study, the optimum sucrose concentration for in vitro pollen germination and pollen tube growth in rice cultivars was 20% (w/v). The low and high sucrose concentration accelerates pollen grain bursting. The 0% sucrose concentration accelerated pollen grain bursting due to turgor pressure as shown in previous studies (Benkert et al. 1997; Pietruszka 2013), whereas the high sucrose concentration lowered the pollen germination rate by inhibiting pollen germination and pollen tube growth because of plasmolysis (Lin et al. 2017). Plasmolysis due to normal water loss resulted in reduced pollen germination rate (Barnabás 1985). In the present study, sucrose concentration and plasmolysis could affect pollen germination. Assessing the incubation period and temperatures required for pollen germination and pollen tube elongation is crucial for in vitro pollen viability analysis. To validate this, we used fresh mature pollen from three rice cultivars. Under favorable in vitro conditions, pollen can germinate and develop pollen tubes in the pollen germination medium.

The pollen tube bursting trend was similar to that of the germination rate; the longer the pollen was incubated, the more pollen tubes burst. This indicates a positive association between the pollen germination incubation period and pollen tube bursting. Weather and other internal factors may also affect pollen grain germination (Pacini and Dolferus 2019; Rang et al. 2011). Regardless of the time taken to collect pollen samples, some pollen did not germinate. This may have been caused by weather changes, especially variations in light, which facilitated the initiation of anthesis.

We tested pollen culture temperatures at 25, 27 and 30°C in ‘Nanatsuboshi’. The results indicate that 25 and 27°C were optimal temperatures, with no significant differences in pollen germination. However, no pollen germination was observed at 30°C. These findings suggest that incubation temperatures are optimal at 25 to 27°C for in vitro pollen germination of Japanese rice cultivars. The plant materials used in this study were maintained in the greenhouse without a heating system during summer, and the environment might affect pollen germination. When testing three cultivars ‘Nanatsuboshi’, ‘Nipponbare’, and ‘Kitaake’, different results were observed in pollen germination (Figure 3A–C) and pollen tube growth (Figure 3D–F) at 25°C but similar trends were observed across all cultivars. Therefore, similar trends would also be observed at 27°C.

In summary, we developed an efficient optimized protocol for high pollen germination, pollen tube growth and pollen tube bursting assessments in Japanese rice pollen. These findings can be used to guide the development of procedures for the in vitro pollen germination in other rice cultivars. As rice pollen seems to easily lose viability, we suggest that more studies be conducted to explore the proper method of short- or long-term storage of rice pollen grains for future use to enable researchers to test pollen viability at any given time.
